# Regulation of Early Cartilage Destruction in Inflammatory Arthritis by Death Receptor 3

**DOI:** 10.1002/art.38770

**Published:** 2014-09-26

**Authors:** Eddie C Y Wang, Zarabeth Newton, Olivia A Hayward, Stephen R Clark, Fraser Collins, William V Perks, Ravinder K Singh, Jason P Twohig, Anwen S Williams

**Affiliations:** Cardiff Institute of Infection & Immunity, Cardiff University School of Medicine, CardiffWales, UK

## Abstract

**Objective:**

To investigate the role of death receptor 3 (DR-3) and its ligand tumor necrosis factor–like molecule 1A (TL1A) in the early stages of inflammatory arthritis.

**Methods:**

Antigen-induced arthritis (AIA) was generated in C57BL/6 mice deficient in the DR-3 gene (DR3^−/−^) and their DR3^+/+^ (wild-type) littermates by priming and intraarticular injection of methylated bovine serum albumin. The joints were sectioned and analyzed histochemically for damage to cartilage and expression of DR3, TL1A, Ly-6G (a marker for neutrophils), the gelatinase matrix metalloproteinase 9 (MMP-9), the aggrecanase ADAMTS-5, and the neutrophil chemoattractant CXCL1. In vitro production of MMP-9 was measured in cultures from fibroblasts, macrophages, and neutrophils following the addition of TL1A and other proinflammatory stimuli.

**Results:**

DR3 expression was up-regulated in the joints of wild-type mice following generation of AIA. DR3^−/−^ mice were protected against cartilage damage compared with wild-type mice, even at early time points prior to the main accumulation of Teff cells in the joint. Early protection against AIA in vivo correlated with reduced levels of MMP-9. In vitro, neutrophils were major producers of MMP-9, while neutrophil numbers were reduced in the joints of DR3^−/−^ mice. However, TL1A neither induced MMP-9 release nor affected the survival of neutrophils. Instead, reduced levels of CXCL1 were observed in the joints of DR3^−/−^ mice.

**Conclusion:**

DR-3 drives early cartilage destruction in the AIA model of inflammatory arthritis through the release of CXCL1, maximizing neutrophil recruitment to the joint and leading to enhanced local production of cartilage-destroying enzymes.

Death receptor 3 (DR-3; also known as TRAMP, lymphocyte-associated receptor of death, WSL-1, Apo-3, TR3, and tumor necrosis factor receptor superfamily member 25 [TNFRSF25]) is the closest relative to TNFR type I (TNFRI; TNFRSF1), one of the major ligands for TNFα, the archetypal “master regulator” of inflammation (1). Like TNFRI, DR-3 has an intracellular death domain that can recruit downstream effectors of apoptosis (2–7) but can also activate the transcription factor NF-κB, inducing immune activation and differentiation (8,9). It has a single TNFSF ligand, TNF-like protein 1A (TL1A; TNFSF15) (9,10), that is closely related in structure to TNFα (11).

In recent years, the DR-3/TL1A axis has emerged as a key regulator of inflammation and autoimmunity in its own right, with in vivo studies of transgenic mice deficient for DR-3 or TL1A and those overexpressing TL1A or dominant-negative forms of DR-3 providing compelling evidence for an essential role of the DR-3/TL1A axis in many models of inflammatory and autoimmune disease (12–22). In contrast to TNFRI, much of the function of DR-3 has been attributed to its expression on T cells and natural killer T cells and its role in driving the accumulation or maintenance (23) of Teff cell numbers at sites of pathology, irrespective of their lineage. Consistent with this, DR-3 has also been shown to be essential for the development of efficient T cell immunity to certain bacterial and viral pathogens (24,25) and, in some cases, becoming essential for host survival (25). DR-3 expression is not restricted to lymphoid cells. In cells of myeloid lineage, in vitro DR-3 signaling can influence cytokine release (26) and myeloid cell differentiation (27), while in nonhematopoietic cells in vivo, DR-3 is expressed on neurones controlling neuronal innervation (28) or can be triggered on tubular epithelial cells to regulate responses to renal inflammation and injury (29,30).

Rheumatoid arthritis (RA) is a chronic inflammatory disease characterized by immune cell infiltration into the joints, which eventually leads to destruction of cartilage and bone. Proinflammatory cytokines such as TNFα and interleukin-6 (IL-6) are critical for controlling the pathogenic process (31,32). A role for the DR-3/TL1A pathway has been proposed in RA, because the frequency of DR-3 gene duplication is higher in patients with RA compared with healthy individuals (33). In contrast, TL1A levels are increased in RA serum (34), synovial fluid, and synovial tissue, and the expression of TL1A can be induced by immune complex–stimulated monocytes in RA (35). This has been borne out in in vivo studies demonstrating that DR3^−/−^ mice with experimental inflammatory arthritis are resistant to bone erosion, while treatment with antagonistic antibodies was protective in wild-type (WT) mice (21). Mechanistically, this effect has been attributed to the control of multiple late events in the arthritis disease process, from effector Th17 cell development (36) and differentiation of macrophages into osteoclasts (21) to the potential action of TL1A on osteoblasts (37).

In the current study, we investigated the in vivo role of the DR-3/TL1A pathway in early events in antigen-induced arthritis (AIA), uncovering previously overlooked functions of this proinflammatory pathway that have an impact on neutrophil recruitment and cartilage degradation.

## MATERIALS AND METHODS

### Animals

DR3^−/−^ mice and their age-matched DR3^+/+^ (WT) littermates (ages 6–12 weeks) were used in the experiments; these mice were derived from a mouse colony with heterozygous DR-3 expression that was founded from mice provided by Cancer Research UK (38). AIA was generated in male mice only. All procedures were approved by the local Research Ethics Committee and were performed in accordance with Home Office–approved licenses PPL 30/1999, 30/2361, and 30/2480.

### Generation of murine AIA

AIA was generated as previously described (39). Briefly, mice were subcutaneously immunized with 1 mg/ml of methylated bovine serum albumin (mBSA) and Freund's complete adjuvant (CFA), in conjunction with an intraperitoneal injection of heat-inactivated *Bordetella pertussis* toxin. A booster immunization of BSA and CFA was administered 1 week later. Arthritis was induced in the hind right knee joint via an intraarticular injection of 10 mg/ml mBSA, given 21 days after the initial immunizations.

### Assessment of cartilage degradation

The mice were killed on day 3 or day 21 after the induction of arthritis, for assessment of inflammatory and pathologic changes within the joint. For in vitro assays, whole murine patellae were incubated with neutrophil lysates for 3 days. All samples were then fixed in neutral buffered formalin and decalcified with formic acid (10%) for 2 weeks at 4°C, prior to embedding in paraffin. Serial sections (7 μm thick) were obtained, deparaffinized, and stained with Safranin O–fast green or toluidine blue, both of which are cationic stains that dye the acidic proteoglycan present in cartilage tissue red or purple. Total cartilage depth was then measured under 40× magnification using a line-graduated scale. The depth of cartilage depletion was determined by measuring to the “tideline” created by the absence of Safranin O–fast green or toluidine blue staining (Figure 2), and a percentage relative to the total cartilage depth was generated. Five points on the femoral head were measured to give an overall score for each joint.

### Immunohistochemical analysis

Expression of the target ligand/receptor was detected using anti-rat, anti-rabbit, or anti-goat horseradish peroxidase (HRP)–diaminobenzidine (DAB) staining kits (R&D Systems), depending on the primary antibody, and according to the manufacturer's instructions. Briefly, sections were rehydrated, and endogenous peroxidase activity was blocked. Antigen unmasking was achieved by incubating the sections in prewarmed trypsin–EDTA (0.1%) in phosphate buffered saline (PBS) for 30 minutes at 37°C. Following the blocking steps, the sections were incubated overnight with 4 μg/ml of rat anti–Ly-6G (Invitrogen), goat anti–matrix metalloproteinase 9 (anti–MMP-9; Santa Cruz Biotechnology), rabbit anti-CXCL1 (Clontech), goat biotinylated anti–DR-3 (R&D Systems), or isotype controls diluted in PBS followed by biotinylated secondary antibody, according to the manufacturers' instructions. Sections were counterstained with hematoxylin, dehydrated, and mounted in DPX. Positive staining was visualized using a streptavidin–HRP conjugate and DAB chromogen that stained positive areas brown. Images were captured using a digital camera (Olympus N457 or Canon EOS 100D), and the proportion of brown pixels within a particular area was measured using Adobe Photoshop CS3.5. Five randomly selected areas were used to generate scores for each sample.

### In vitro cell culture

Human monocytes were obtained from peripheral blood using density-gradient centrifugation to purify mononuclear cells, followed by isolation with anti-CD14 microbeads according to the manufacturer's instructions (Miltenyi Biotec). Macrophages were then generated by 7-day culture in RPMI 1640 supplemented with 10% heat-inactivated fetal calf serum and macrophage colony-stimulating factor (20 ng/ml; R&D Systems). Human neutrophils, skin, and synovial fibroblasts were isolated as previously described (40–42). Ethics approval for all human experiments was obtained from the Bro Taf Health Authority (Cardiff, Wales, UK) prior to commencement of the study. Murine bone marrow–derived macrophages were generated from bone marrow extracted from the femurs of DR3^−/−^ and WT mice, as previously described (21). Cells were cultured with or without recombinant TL1A or murine soluble DR-3 (R&D Systems) at the concentrations indicated, sometimes with additional stimuli such as interferon-γ (IFNγ) (26), lipopolysaccharide, or antigen/antibody complexes (35). Supernatants were collected over a 24-hour period, and the concentrations of enzymes, chemokines, or cytokines were measured as indicated.

### Enzyme-linked immunosorbent assays (ELISAs)

ELISAs for murine CXCL1 and human MMP-9 were performed according to the instructions of the manufacturer (R&D Systems).

### Statistical analysis

Cartilage degradation and staining readouts were percentages; therefore, Mann-Whitney nonparametric U tests were used for statistical analysis. Student's *t*-tests were used in the analyses of protein concentrations determined by ELISAs. Analyses were performed using GraphPad Prism version 4. *P* values less than or equal to 0.05 were considered significant, and *P* values less than or equal to 0.01 were considered highly significant.

## RESULTS

### DR-3 expression in inflamed joints

**Figure 1:**
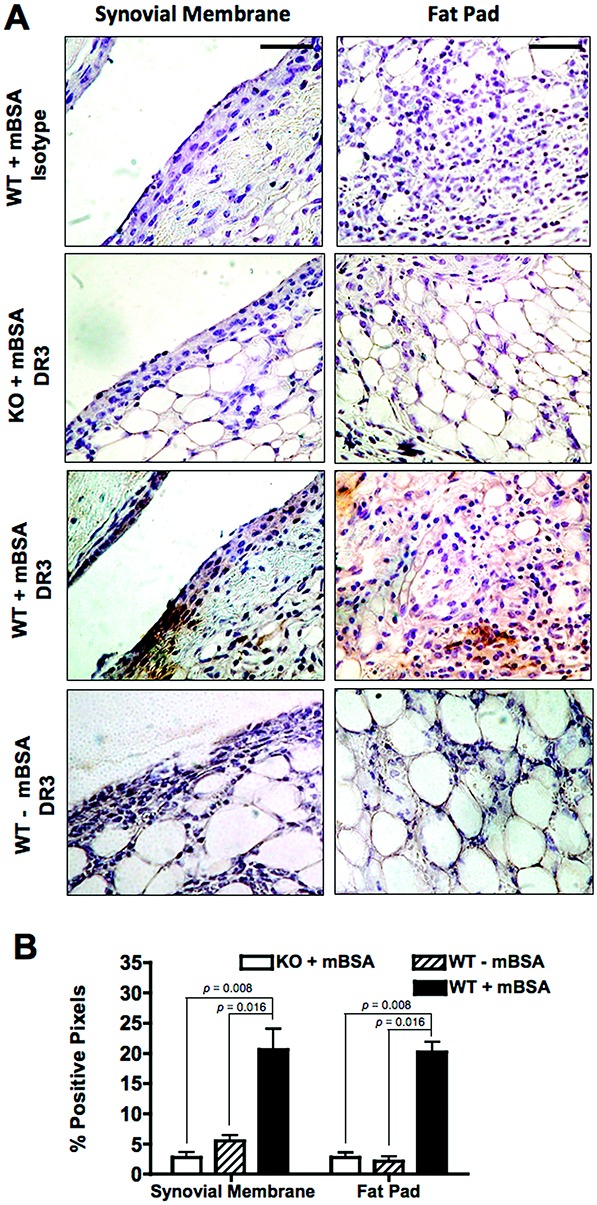
Death receptor 3 (DR-3) expression in the joints of mice with antigen-induced arthritis. Arthritis was induced in DR3-knockout (DR3-KO; DR3^−/−^) mice and their DR3^+/+^ (wild-type [WT]) littermates, and the joints were prepared, sectioned, and stained for DR-3 as described in Materials and Methods. Antigen (methylated bovine serum albumin [mBSA]) was administered into the right knee to induce localized inflammatory arthritis. A, Representative high-magnification (40×) photomicrographs showing (from top to bottom) isotype staining in a right knee section from a WT mouse, anti– DR-3 staining in a right knee section from a DR3^−/−^ mouse, anti–DR-3 staining in a right knee section from a WT mouse, and anti–DR-3 staining in a left knee section (contralateral negative control) from a WT mouse. Bars = 45 μm. B, Quantification of positive staining, as measured by the percentage of positive pixels within a particular area. Values are the mean ± SEM (n = 4–5 mice per group). *P* values were determined by Mann-Whitney U test.

Although the DR-3/TL1A pathway has been shown to be essential in the development of inflammatory arthritis in mice, and that antagonism of this pathway can ameliorate disease (21), relatively little is known about the expression patterns of members of this pathway in the joint. Here, we investigated DR-3 expression early in the inflammatory process by staining joint sections with a polyclonal antibody. As expected, synovial membrane sections from the joints of WT mice showed minimal isotype staining, and synovial membrane sections from the joints of DR3^−/−^ mice showed minimal anti–DR-3 staining (Figure 1A) (mean ± SEM 5.5 ± 1.0% and 2.8 ± 0.8%, respectively). In contrast, strong signals for DR-3 were recorded in synovial membrane sections (20.6 ± 3.5%) and anterior fat pad sections (20.2 ± 1.7%) from the joints of WT mice, 3 days after the generation of AIA (13,21). The DR-3 signal was low or absent in sections obtained from both of these areas in lateral control knees, in which mBSA had not been injected (Figure 1A). These data showed that DR-3 is primarily absent in healthy joints (some low-level expression may be present in the synovial membrane) but is significantly and rapidly increased by the inflammatory process induced by injection of mBSA (Figure 1B).

### Protection against early cartilage degradation in the joints of DR3^−/−^ mice

**Figure 2:**
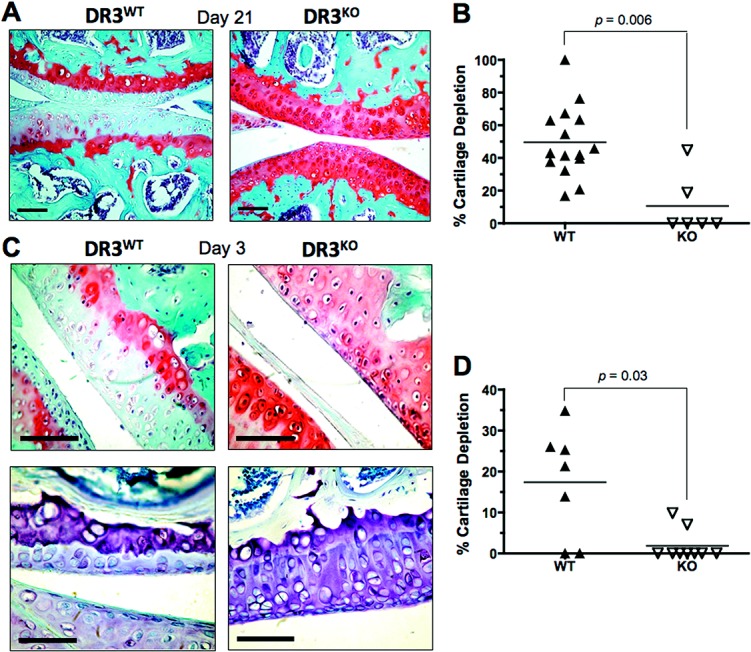
Cartilage depletion in the joints of mice with antigen-induced arthritis (AIA). Arthritis was induced in WT and DR3^−/−^ mice, and the joints were prepared, sectioned, and stained as described in Materials and Methods. A, Representative Safranin O–fast green–stained joint sections from WT and DR3^−/−^ mice, 21 days after generation of AIA. B, Quantification of cartilage depletion in WT and DR3^−/−^ mice on day 21. C, Representative Safranin O–fast green–stained (top row) and toludine blue–stained (bottom row) joint sections from WT and DR3^−/−^ mice, 3 days after generation of AIA. D, Quantification of cartilage depletion in WT and DR3^−/−^ mice on day 3, as measured by Safranin O–fast green staining. In A and C, bars = 60 μm. In B and D, each data point represents a single mouse; horizontal lines show the mean. *P* values were determined by Mann-Whitney U test. See Figure 1 for other definitions.

To examine the functional significance of this increase in DR-3 expression, we investigated cartilage degradation at both early (day 3) and late (day 21) time points following generation of AIA. Consistent with a previous report (21), DR3^−/−^ mice showed significant protection against cartilage destruction compared with their WT littermates on day 21 (mean ± SEM 11 ± 7% versus 50 ± 6%; *P* = 0.006), as measured by proteoglycan staining with Safranin O–fast green (Figures 2A and B). Unexpectedly, this pattern was also observed early in the inflammatory process on day 3 after generation of AIA (17 ± 5% in WT mice and 2 ± 1% in DR3^−/−^ mice; *P* = 0.03), as measured by staining with Safranin O–fast green or toluidine blue (Figures 2C and D). Thus, the DR-3/TL1A pathway contributes to the development of early pathologic features of inflammatory arthritis prior to exerting an effect on Teff cell development and osteoclastogenesis in murine models of inflammatory arthritis (21,36).

### Concentrations of MMP-9 and ADAMTS-5 in the joints of DR3^−/−^ mice early in the course of AIA

**Figure 3:**
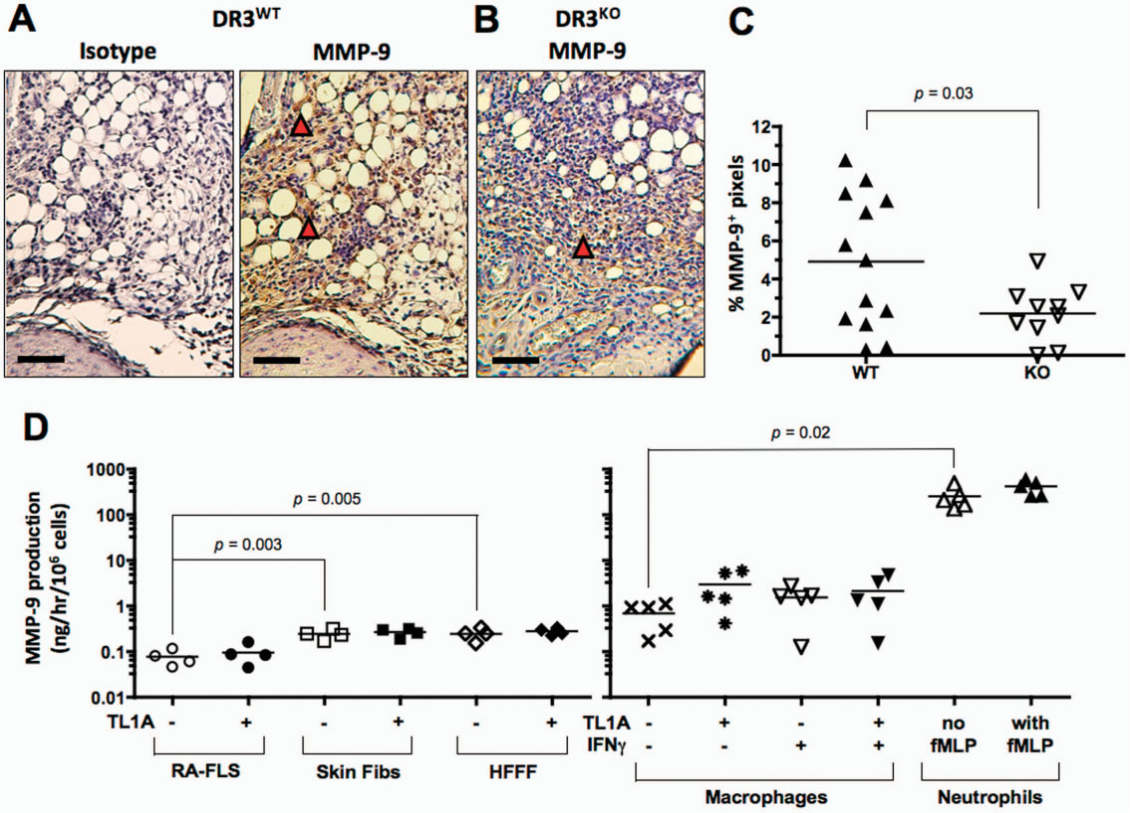
Matrix metalloproteinase 9 (MMP-9) expression in the joints of mice with antigen-induced arthritis and MMP-9 production in vitro. Arthritis was induced in WT mice and DR3^−/−^ mice, and the joints were prepared, sectioned, and stained as described in Materials and Methods. A and B, Representative joint sections from a WT mouse (A) and a DR3^−/−^ mouse (B, 3 days after induction of arthritis, stained for MMP-9. Arrowheads highlight areas of positive brown staining. Bars = 60 μm. C, Quantification of MMP-9 expression in WT and DR3^−/−^ mice. D, MMP-9 production in cultures of rheumatoid arthritis fibroblast-like synoviocytes (RA FLS), healthy skin fibroblasts (fibs), human fetal foreskin fibroblasts (HFFF), macrophages, and neutrophils treated with the indicated stimuli. In C and D, each symbol represents a single mouse (C) or a single subject (D); horizontal lines show the mean. *P* values were determined by Mann-Whitney U test (C) and Student's *t*-test (D). TL1A = tumor necrosis factor–like molecule 1A; IFNγ = interferon-γ (see Figure 1 for other definitions).

In an attempt to determine how DR-3 so rapidly contributes to joint degradation, we investigated the level of cartilage-destroying enzymes within the joints of DR3^−/−^ and WT mice in early AIA. MMP-9, a gelatinase that degrades type IV and type V collagen and has an established role in cartilage degradation during RA (43), was chosen because of previous reports that MMP-9 release could be induced from the myeloid cell line THP-1 in vitro by either crosslinking of DR-3 (44) or the action of IFNγ and TL1A (26). Consistent with the observed reductions in cartilage depletion, MMP-9 levels were significantly reduced in the joints of DR3^−/−^ mice with AIA (mean ± SEM 2.3 ± 0.4%) compared with the levels in WT mice (4.4 ± 0.7%; *P* = 0.03) 3 days after the initiation of AIA (Figures 3A and C). This was primarily attributable to the presence of MMP-9 within infiltrating cells in the fat pad (Figures 3A and C), but MMP-9 was also detected in chondrocytes from the joints of WT and DR3^−/−^ mice (additional information is available from the corresponding author). In contrast, the levels of ADAMTS-5, the major aggrecanase in mouse cartilage (45), in the joints of DR3^−/−^ mice were not different from the levels in WT mice (additional information is available from the corresponding author). Therefore, at this time point (day 3), levels of MMP-9, but not ADAMTS-5, were dependent on the presence of DR-3.

### Neutrophils as a major source of MMP-9

In order to determine the potential source of DR-3–dependent MMP-9, cell lines representing stromal and infiltrating cell types within the inflamed joint were established and tested for MMP-9 production in response to TL1A. These included fibroblasts derived from multiple sources (RA synovium, healthy skin, or fetal foreskin), macrophages, and neutrophils. As expected, fibroblasts produced only small amounts of MMP-9 (on a per-cell basis), with skin fibroblasts and fetal foreskin fibroblasts producing significantly more than RA synovial fibroblasts (mean ± SEM 0.15 ± 0.01, 0.15 ± 0.02, and 0.05 ± 0.01 ng/hour/million cells, respectively). In contrast, primary macrophages produced ∼20 times more MMP-9 (3.1 ± 0.9 ng/hour/million cells) than fibroblasts, and neutrophils generated ∼800 times more MMP-9 (126 ± 31 ng/hour/million cells) than fibroblasts (Figure 3D).

**Figure 4:**
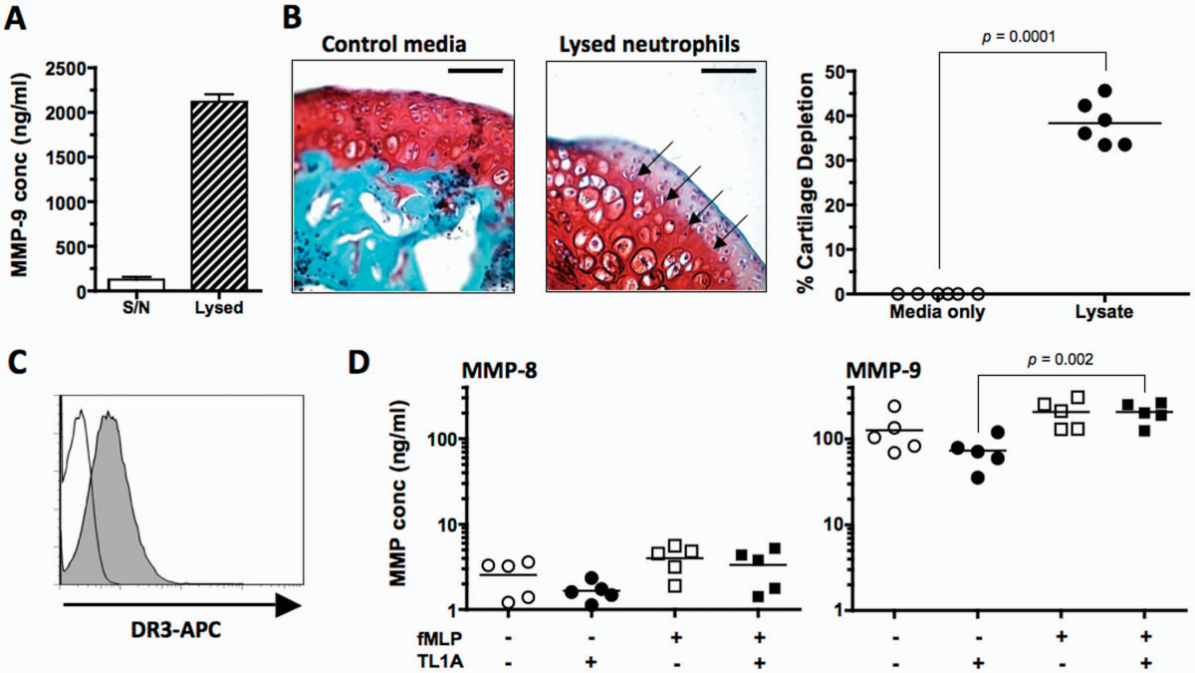
Matrix metalloproteinase 9 (MMP-9) production by neutrophils. A, Concentration (conc) of MMP-9 in neutrophil culture supernatants (S/N) and lysates. Values are the mean ± SEM. B, Left, Representative whole murine patellae sections incubated with control media or neutrophil lysates. Arrows indicate the tidemark used to determine cartilage degradation. Bars = 60 μm. Right, Percentage of cartilage degradation. C, Histogram showing death receptor 3 (DR-3) expression on neutrophils, as determined by flow cytometric analysis. D, MMP-8 and MMP-9 production by neutrophils following in vitro activation. In B and D, each data point represents a single culture; horizontal lines show the mean. *P* values were determined by Mann-Whitney U test (B) or Student's *t*-test (D). TL1A = tumor necrosis factor–like molecule 1A; APC = allophycocyanin.

Although this production was significant, neutrophils contained even larger (17-fold) intracellular stores of MMP-9, as shown by testing lysed cultures by ELISA (Figure 4A). Such lysates were also highly capable of degrading articular cartilage in vitro (Figure 4B). However, although DR-3 was observed on the surface of neutrophils (Figure 4C), and general activation using fMLP significantly increased the production of MMP-9 by neutrophils, TL1A did not stimulate release of MMP-9 (Figure 4D). In addition, TL1A and fMLP activation had no significant effect on the release of the neutrophil collagenase MMP-8, which was observed at concentrations ∼50-fold less than those of MMP-9 in neutrophil culture supernatants (Figure 4D). Indeed, TL1A did not increase MMP-9 release from any of the cultured cells examined (Figure 3D). Thus, although neutrophils were the likeliest source of cartilage-depleting MMP-9 in the joints of mice with AIA, TL1A does not appear to elevate levels of MMP-9 by directly inducing production.

### Impaired neutrophil infiltration into the joints of DR3^−/−^ mice early in the course of AIA

**Figure 5:**
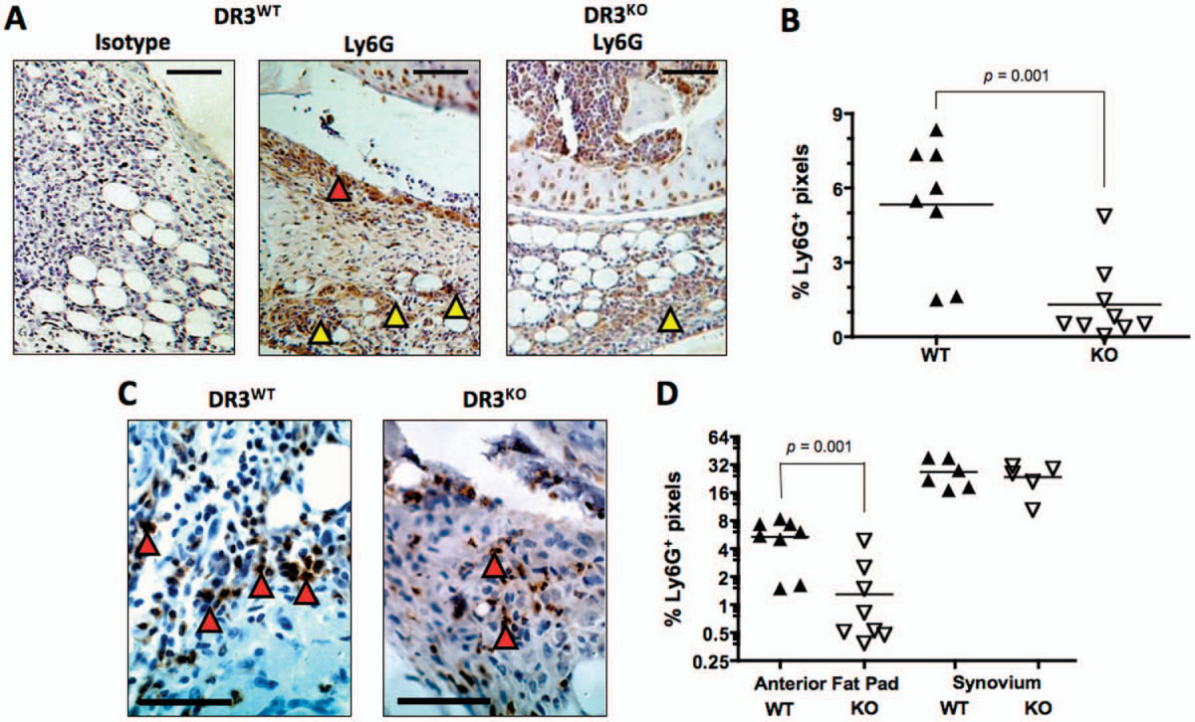
Expression of the neutrophil marker Ly-6G in the joints of mice with antigen-induced arthritis. Arthritis was induced in WT and DR3^−/−^ mice, and the joints were prepared, sectioned, and stained as described in Materials and Methods. A, Representative low-magnification photomicrographs of joint sections from WT and DR3^−/−^ mice stained for Ly-6G, 3 days after induction of arthritis. Arrowheads highlight staining in the synovial membrane (red) or fat pad (yellow). B, Quantification of Ly-6G expression in the joints of WT and DR3^−/−^ mice. C, Representative high-magnification photomicrographs of fat pad sections from the joints of WT and DR3^−/−^ mice. Arrowheads highlight staining of infiltrating cells. D, Quantification of Ly-6G expression in fat pad and synovial membrane sections obtained from the right knees of WT and DR3^−/−^ mice. Bars in A and C = 60 μm. In B and D, each data point represents a single mouse; horizontal lines show the mean. *P* values were determined by Mann-Whitney U test. See Figure 1 for definitions.

We hypothesized that DR-3 could control early pathologic changes in the joint by increasing the number of infiltrating innate effector cells bearing MMP-9, which would include macrophages and neutrophils. Previous studies have indicated that there is no difference between the level of macrophages in the joints of DR3^−/−^ and WT mice with AIA, early or late in the disease course, as measured by F4/80 staining (21). The predominant cell type involved in early infiltration into the joints of mice with AIA are neutrophils, which can be observed as soon as 6 hours after antigen injection (46). Thus, we stained the joints of mice with AIA that were killed on day 3 for the neutrophil marker Ly-6G. The joints of DR3^−/−^ mice showed significantly less Ly-6G staining compared with their WT counterparts (mean ± SEM 1.3 ± 0.5% versus 5.3 ± 0.9%; *P* = 0.001) (Figures 5A and B), and this was primarily associated with cellular infiltration into the fat pad (Figures 5C and D). Thus, the accumulation or maintenance of neutrophil numbers in the joint early after the generation of AIA was dependent on DR-3 expression.

### Reduced expression of the neutrophil chemoattractant CXCL1 in the joints of DR3^−/−^ mice

**Figure 6:**
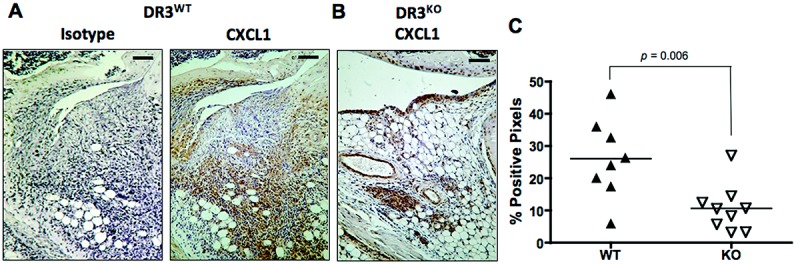
CXCL1 expression in the joints of mice with antigen-induced arthritis. Arthritis was induced in WT and DR3^−/−^ mice, and the joints were prepared, sectioned, and stained as described in Materials and Methods. A and B, Representative joint sections from a WT mouse (A) and a DR3^−/−^ mouse (B), 3 days after induction of arthritis, stained for CXCL1. Bars = 60 μm. C, Quantification of CXCL1 expression in WT and DR3^−/−^ mice. Each data point represents a single mouse; horizontal lines show the mean. *P* values were determined by Mann-Whitney U test. See Figure 1 for definitions.

Several potential mechanisms could explain the reduced expression of CXCL1 in the joints of DR3^−/−^ mice. The most obvious, considering DR-3 contains a death domain, is an alteration in neutrophil survival. However, in vitro experiments indicated that TL1A had no significant effect on neutrophil death, as measured by staining with fluorescein isothiocyanate–labeled annexin V/7-aminoactinomycin D and flow cytometric evaluation, with or without activating stimuli (additional information is available from the corresponding author). Another possible explanation is that DR-3 controlled neutrophil recruitment. A number of chemokines have been reported to attract neutrophils, but the release of human IL-8 from the macrophage-like cell line THP-1 has previously been shown to be triggered in response to TL1A following IFNγ priming (47). We therefore stained the joints of mice with AIA for the murine ortholog of IL-8, CXCL1 (also known as murine keratinocyte-derived chemokine). DR3^−/−^ mouse joints showed significantly less staining for CXCL1 than joints from WT mice (mean ± SEM 11 ± 2% and 26 ± 4%, respectively; *P* = 0.006) (Figures 6A and C). These data are consistent with the hypothesis that a reduction in production of neutrophil attractants such as CXCL1, rather than any affect on survival or lifespan, causes the decrease in neutrophil infiltration in DR3^−/−^ mice early in the inflammatory process of AIA.

## DISCUSSION

The DR-3/TL1A pathway has recently emerged as a potential therapeutic target in inflammatory arthritis, the antagonism of which could impair the mechanisms that are controlled by this pathway. These include the development of effector CD4+ Th17 cells (36), macrophage differentiation into osteoclasts (21), and osteoblast function (37), all of which influence late events in the inflammatory arthritis disease process through an impact on bone turnover. Here, we show that DR-3 also controls early stages of the pathogenic process by regulating the initial damage to cartilage that occurs prior to the events described above.

To our knowledge, DR-3 expression patterns in the joint have not been previously described, and only one study has shown the presence of its TNFSF ligand TL1A in the joints of patients with RA (35). In the current study, we show that DR-3 is present only at low levels in unchallenged contralateral joints but is up-regulated following injection of arthritis-inducing antigen (Figure 1). The simplest interpretation of these observations is that local antigen-driven signals induce up-regulation of DR-3; however, a degree of caution is required. The strongest DR-3 signals came from the areas just below the synovial membrane and from within the fat pad, but these signals localized to the same areas that stained infiltrating neutrophils using Ly-6G (Figure 4). The fact that DR-3 was detected on the surface of human neutrophils (Figure 4) and has also been observed on macrophage-like cell lines and primary macrophages (26), means the extent to which increasing DR-3 signals can be attributed to induction of expression on stromal cells versus its surface expression on infiltrating cells cannot yet be judged. Interestingly, a more general diffuse signal throughout the joints of WT mice with AIA was also observed (Figure 1) and would be consistent with the presence of soluble DR-3.

At least 3 murine splice variants have been described, including a soluble form lacking a transmembrane region (48), the expression of which is differentially regulated by activation (7,25). The function of these different splice variants is still poorly understood, but soluble DR-3 should buffer the action of TL1A. In mice, this may be particularly significant, because there is no known murine homolog for human decoy receptor 3 (DcR-3), which is described as an additional soluble decoy ligand for 3 TNFSF members (TL1A, FasL, and LIGHT) (49), and its level has also been shown to be increased in the serum of patients with RA (34). Human DcR-3 also binds murine TL1A, FasL, and LIGHT (49), and it is interesting that its systemic application in a murine model of collagen-induced arthritis (CIA) resulted in amelioration of disease associated with inhibition of effector CD4+ T cells and B cells (50). This is consistent with studies by our group and other investigators demonstrating the role of DR-3 in AIA and CIA (21,36), as is the ability of DcR-3 to inhibit osteoclastogenesis in vitro (51), but neither study determined the dominant pathway(s) through which DcR-3–dependent inhibition occurred. These differences between species and the complexity of TNFSF and TNFRSF interactions are clearly areas that should be studied further in inflammatory diseases such as RA.

Although many MMPs, including MMP-1, MMP-2, MMP-3, MMP-9, and MMP-13, have been associated with the destruction of cartilage, tendon, and bone in RA (43), the current study focused on MMP-9 because of several previous in vitro studies demonstrating its TL1A-driven release from macrophage-like cell lines (26,44). MMP-9 is primarily a gelatinase but also targets type IV collagen and is believed to further degrade extracellular matrix after the action of type I and II collagenases such as MMP-1 and MMP-13. Increasing levels of serum MMP-9 correlate with RA severity (52,53), while MMP-9–deficient mice show resistance to antibody-induced arthritis (54). Our data showed for the first time that the absence of DR-3 is associated with a significant decrease in MMP-9 expression at very early stages in the development of AIA. Interestingly, the absence of DR-3 was not associated with a change in the levels of ADAMTS-5, an aggrecanase responsible for cartilage degradation in osteoarthritis (55), suggesting that DR-3 signaling differentially regulates the levels of some (e.g., MMP-9) but not other cartilage-destroying enzymes at this early time point in the AIA process. Further study at later time points, when more effector Th17 cells would be present in the joint, would be required to determine whether the reported synergistic induction of ADAMTS-5 from macrophages by TL1A and IL-17 occurs in AIA (56).

We also discovered that neutrophils produced ∼40 times more MMP-9 in culture on a per-cell basis than macrophages, with an additional capacity to produce ≥600 times more MMP-9 due to high intracellular stores (Figures 3 and 4). Neutrophils also produce MMP-8, although this collagenase was generated at ∼50-fold lower concentrations than MMP-9 in our in vitro cultures and was not significantly induced by activation or exogenous TL1A (Figure 4). This does not preclude a role for MMP-8 in cartilage destruction in inflammatory arthritides, but suggests that there may be a hierarchy of MMP production from neutrophils, several of which could contribute to cartilage destruction.

These data are consistent with the results of several studies showing that neutrophils are a primary source of MMP-9 in diseases requiring breakdown of tissue, such as coronary heart disease (57) or stroke (58), although their potential to contribute significantly to MMP-9 levels in the inflamed joint has not previously been described. Instead, it has been suggested that macrophages are the primary source of MMP-9 in RA (59,60). The potential role of neutrophils in the early pathogenesis of RA seems to have mostly been ignored, probably because patients often present with later-stage disease, when joint damage has already occurred and swelling has resolved. Historically, however, it has been estimated that the turnover of neutrophils is extremely high in inflamed joints (61), with the main role for neutrophils in models of inflammatory arthritis being attributed to the supply of proinflammatory leukotrienes (62,63).

Intriguingly, we failed to reproduce the previous in vitro findings of TL1A-driven MMP-9 release, although this may in part have been attributable to our use of primary cells, which may require additional signals for priming. Kang and colleagues demonstrated these effects using THP-1 cells, which also required priming with interferon-γ (26). Instead, the role of the DR-3/TL1A pathway at this early stage in the development of inflammatory arthritis in murine AIA seems to be the production of chemokines that attract neutrophils into the inflamed joint. In humans, IL-8 (CXCL8) is considered to be the primary neutrophil chemoattractant and has itself been reported to induce MMP-9 release (64,65). Mice, however, do not have a CXCL8 homolog, with CXCL1 (keratinocyte-derived chemokine) being considered the murine functional ortholog of IL-8. To our knowledge, there are no studies investigating whether CXCL1 can trigger MMP-9 release, but it is interesting to note that studies of human neutrophils have suggested that signaling through CXCR2, and not CXCR1, induces IL-8–dependent MMP-9 release (65).

CXCL1 levels were reduced in the absence of DR-3 (Figure 6), but to date, we have been unable to confirm the exact source of DR-3–dependent CXCL1 in the joints of mice with AIA. Neutrophils, macrophages, and epithelial cells have all been reported to release CXCL1 (66,67). The pattern of more Ly-6G–positive neutrophils in the fat pad but not around the synovial membranes in the joints of WT mice (Figure 5) would be consistent with a DR-3–independent source of CXCL1 from stromal cells, with further CXCL1 being provided by infiltrating cells in a DR-3–dependent manner. However, our in vitro experiments in bone marrow–derived macrophages from DR3^−/−^ and WT mice have shown both increases and decreases in DR-3–dependent CXCL1 production triggered by the addition of TL1A (data not shown). This is likely to reflect the intrinsic plasticity of macrophages, coupled with the effects of DR-3/TL1A signaling impacting on target cells at different stages of differentiation. This has been previously observed with CD4+ T cells, in which TL1A inhibits the differentiation of naive cells to Th17 cells but maintains the numbers of these Teff cells once they are committed to the IL-17–producing lineage (23).

The description of a reduction in the accumulation of neutrophils in the joints of DR3^−/−^ mice 3 days after the generation of AIA is novel. Previous studies have suggested that cellular infiltration at this time point was not different between DR3^−/−^ mice and WT mice (21), but in those studies only macrophage infiltration was investigated in any detail, using staining for F4/80. Here, we used Ly-6G as a stain, with the microscopic study of Ly-6G–positive cells showing morphologic characteristics of neutrophils (data not shown). In so doing, we also identify neutrophils as a major source of MMP-9 early in the course of AIA and highlight a novel function for DR-3, namely, the recruitment of neutrophils to inflamed joints. It is clear that the DR-3/TL1A pathway regulates multiple functions relating to the development of inflammatory and autoimmune disease, and further study is required to determine how antagonism of this pathway may be used as a potential treatment in the future.

## AUTHOR CONTRIBUTIONS

All authors were involved in drafting the article or revising it critically for important intellectual content, and all authors approved the final version to be published. Dr. Wang had full access to all of the data in the study and takes responsibility for the integrity of the data and the accuracy of the data analysis.

**Study conception and design.** Wang, Newton, Williams.

**Acquisition of data.** Wang, Newton, Hayward, Clark, Collins, Perks, Singh, Twohig, Williams.

**Analysis and interpretation of data.** Wang, Newton, Williams.
